# StreptomeDB 3.0: an updated compendium of streptomycetes natural products

**DOI:** 10.1093/nar/gkaa868

**Published:** 2020-10-13

**Authors:** Aurélien F A Moumbock, Mingjie Gao, Ammar Qaseem, Jianyu Li, Pascal A Kirchner, Bakoh Ndingkokhar, Boris D Bekono, Conrad V Simoben, Smith B Babiaka, Yvette I Malange, Florian Sauter, Paul Zierep, Fidele Ntie-Kang, Stefan Günther

**Affiliations:** Institute of Pharmaceutical Sciences, Albert-Ludwigs-Universität Freiburg, Hermann-Herder-Straße 9, D-79104 Freiburg, Germany; Institute of Pharmaceutical Sciences, Albert-Ludwigs-Universität Freiburg, Hermann-Herder-Straße 9, D-79104 Freiburg, Germany; Institute of Pharmaceutical Sciences, Albert-Ludwigs-Universität Freiburg, Hermann-Herder-Straße 9, D-79104 Freiburg, Germany; Institute of Pharmaceutical Sciences, Albert-Ludwigs-Universität Freiburg, Hermann-Herder-Straße 9, D-79104 Freiburg, Germany; Institute of Pharmaceutical Sciences, Albert-Ludwigs-Universität Freiburg, Hermann-Herder-Straße 9, D-79104 Freiburg, Germany; Department of Organic Chemistry, University of Yaoundé I, P. O. Box 812, Yaoundé, Cameroon; Department of Physics, Higher Teacher Training College, University of Yaoundé I, P. O. Box 47, Yaoundé, Cameroon; Department of Pharmaceutical Chemistry, Martin-Luther-Universität Halle-Wittenberg, Wolfgang-Langenbeck Straße 4, D-06120 Halle (Saale), Germany; Department of Chemistry, University of Buea, P. O. Box 63, Buea, Cameroon; Department of Chemistry, University of Buea, P. O. Box 63, Buea, Cameroon; Institute of Pharmaceutical Sciences, Albert-Ludwigs-Universität Freiburg, Hermann-Herder-Straße 9, D-79104 Freiburg, Germany; Institute of Pharmaceutical Sciences, Albert-Ludwigs-Universität Freiburg, Hermann-Herder-Straße 9, D-79104 Freiburg, Germany; Department of Pharmaceutical Chemistry, Martin-Luther-Universität Halle-Wittenberg, Wolfgang-Langenbeck Straße 4, D-06120 Halle (Saale), Germany; Department of Chemistry, University of Buea, P. O. Box 63, Buea, Cameroon; Institute of Botany, Technische Universität Dresden, Zellescher Weg 20b, D-01217 Dresden, Germany; Institute of Pharmaceutical Sciences, Albert-Ludwigs-Universität Freiburg, Hermann-Herder-Straße 9, D-79104 Freiburg, Germany

## Abstract

Antimicrobial resistance is an emerging global health threat necessitating the rapid development of novel antimicrobials. Remarkably, the vast majority of currently available antibiotics are natural products (NPs) isolated from streptomycetes, soil-dwelling bacteria of the genus *Streptomyces*. However, there is still a huge reservoir of streptomycetes NPs which remains pharmaceutically untapped and a compendium thereof could serve as a source of inspiration for the rational design of novel antibiotics. Initially released in 2012, StreptomeDB (http://www.pharmbioinf.uni-freiburg.de/streptomedb) is the first and only public online database that enables the interactive phylogenetic exploration of streptomycetes and their isolated or mutasynthesized NPs. In this third release, there are substantial improvements over its forerunners, especially in terms of data content. For instance, about 2500 unique NPs were newly annotated through manual curation of about 1300 PubMed-indexed articles, published in the last five years since the second release. To increase interoperability, StreptomeDB entries were hyperlinked to several spectral, (bio)chemical and chemical vendor databases, and also to a genome-based NP prediction server. Moreover, predicted pharmacokinetic and toxicity profiles were added. Lastly, some recent real-world use cases of StreptomeDB are highlighted, to illustrate its applicability in life sciences.

## INTRODUCTION

**Figure 1.**
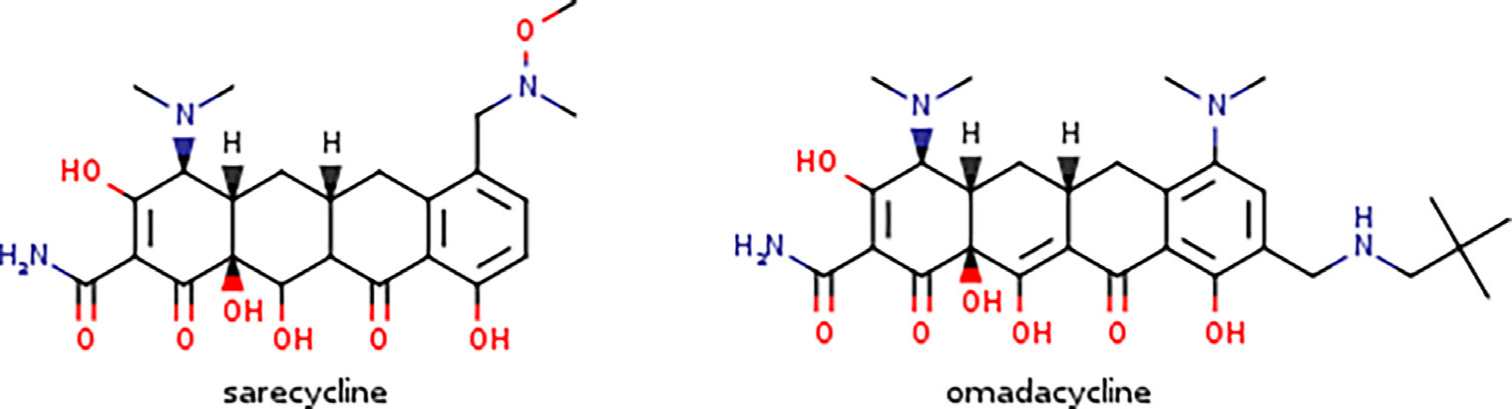
Chemical structures of two recently FDA-approved tetracycline-derived antibiotics.

Microbial pathogens are gradually becoming less sensitive or even fully resistant to the currently available antimicrobials, typically as a result of genetic mutations or the misuse of antimicrobials (1). Consequently, the World Health Organization has classified antimicrobial resistance as an emerging global health threat (2), necessitating the rapid development of new antimicrobials. A recent literature survey by Newmann and Cragg shows that more than half of the antibacterials newly approved over the last four decades (3) are either natural products (NPs) or their synthetic derivatives, collectively known as antibiotics. Remarkably, the vast majority of them stem from streptomycetes, filamentous Gram-positive soil-dwelling bacteria of the genus *Streptomyces* (4). One of the most recent success stories includes the market approval by the US Food and Drug Administration (FDA) in 2018 of two tetracycline-derived third generation antibiotics: sarecycline and omadacycline (Figure 1), displaying narrow- and broad-spectrum antimicrobial activities, respectively (5,6).

Importantly, there is still a huge reservoir of streptomycetes NPs possessing atypical scaffolds, whose therapeutic potentials have not yet been fully investigated. In addition to their broad-spectrum antimicrobial activities, streptomycetes NPs have been reported to display various other types of activity including antiparasitic, anticancer, and anti-inflammatory activities (7). Given the recent advances in computer-aided drug discovery and multi-omics approaches to harness NPs (8,9), a compendium of streptomycetes NPs could serve as a source of inspiration for the rational design of new drugs of various classes.

Initially released in 2012, StreptomeDB is the first public online database enabling the interactive phylogenetic exploration of streptomycetes and their isolated or mutasynthesized NPs (10). Other databases containing microbial NPs include the Natural Products Atlas (11), Antibase (12), and MarinLit (13). StreptomeDB has evolved over the years (version 2.0 (14) was released in 2015) to become a streptomycetes hub for MS/NMR structural dereplication of newly isolated NPs, substructure searching and similarity searching.

Considering the resurgence in NP-inspired drug discovery and the vast number of published articles describing the isolation and/or mutasynthesis of NPs from streptomycetes, we were prompted to update StreptomeDB. In this third release, there are substantial improvements over its forerunners, especially in terms of data content. The retrieval of relevant PubMed-indexed articles and their full-text curation, as well as scaffold decomposition, phylogenetic tree construction and NMR/MS spectral data prediction, were performed in the same manner as described for previous releases (10,14). However, to increase interoperability, StreptomeDB entries were hyperlinked to several other public online resources. Moreover, computationally predicted drug absorption, distribution, metabolism, excretion, and toxicity (ADMET) profiles were added as new features. After summarizing the growth of StreptomeDB over the years, we shall describe recent developments and highlight some recent real-world use cases exemplifying the applicability of StreptomeDB.

## GROWTH OF THE DATABASE

Notable increments in various StreptomeDB releases are summarized in Table 1. About 2500 NPs and 700 producer streptomycetes strains were newly annotated from about 1300 relevant PubMed-indexed articles published in the last five years, since the second release. Accordingly, the number of compound–organism, compound–biosynthesis route and compound–activity relationships have practically doubled, and meanwhile there is a 34% increase in the number of unique scaffolds since the last release. Overall, these statistics reflect the constantly increasing interest of researchers toward the isolation of bioactive molecules from *Streptomyces* species.

**Table 1.** The growth of the StreptomeDB over the years

	StreptomeDB		
Release number	1.0	2.0	3.0
Publication year	2012	2015	2020
Number of compounds	2444	4040	6524
Number of unique scaffolds	–*	4680	6262
Number of organisms (including strains)	1985	2584	3302
Number of compound–organism relationships	4341	6717	10 912
Number of compound–biosynthesis route relationships	307	731	1392
Number of compound–activity relationships	1036	3813	6850
Number of compounds with predicted NMR data	–	3989	6507
Number of compounds with predicted MS data	–	1945	4943
Number of compounds with predicted ADMET properties	–	–	6524
Number of referenced articles	4544	5486	6754

*not yet implemented.

## RECENT DEVELOPMENTS

### Hyperlinks to other online resources

There is a plethora of experimental (bio)chemical data on streptomycetes and their NPs that is available in a number of disparate public databases, which was aggregated in StreptomeDB to facilitate their access to the research community. Also, we added hyperlinks to chemical vendor databases, which may foster the purchase and further investigations on streptomycetes NPs. Accordingly, when available, PubChem Compound ID (CID) (15) and CAS Registry Number (CAS RN^®^) were included. Further, the retrieved CIDs were mapped to several other online resources via the UniChem cross-reference platform (16). UniChem uses the InChI to easily compare various molecular structure encodings of the same compound from different sources in order to establish molecular uniqueness.

These online resources can be subdivided into three categories. Firstly, spectral databases: NMRShiftDB (17) and GNPS (18), containing NMR and MS data, respectively. Secondly, biochemical databases: ChEMBL (19), PDB (20), DrugBank (21), KEGG (22), ChEBI (23), BindingDB (24), ACToR (25), MetaboLights (26), and EPA CompTox Dashboard (27) collectively contain bioactivity data, metabolic data, toxicity data, biosynthetic pathways and protein–ligand cocrystal structures, etc. Thirdly, chemical vendor databases: MolPort (https://www.molport.com/), Selleck (https://www.selleckchem.com/), and ZINC (28). It is worth mentioning that both PubChem (15) and ChEBI (23) also contain links to chemical vendors websites. For GNPS (18) which is not integrated to UniChem, the relevant IDs were extracted from a downloadable JSON file (https://ccms-ucsd.github.io/GNPSDocumentation/api/).

Nowadays, computational methods that bridge the gap between the genomic and chemical space are instrumental in genomics-driven NP discovery (29–31). Within the framework of the ‘Genes to Metabolites’ approach, we hyperlinked streptomycetes strains to the SeMPI web server (32), a genome-based pipeline we developed, that leverages published genomic data to identify encoding biosynthetic gene clusters of isolated NPs and predicts the putative structures of related NPs that could be synthesized by polyketide synthases of type I (32), and nonribosomal peptide synthetases (unpublished data).

### Predicted ADMET properties

Among other physicochemical descriptors, we predicted ADMET properties of all StreptomeDB molecules using the pkCSM web server (33). The prediction of various pharmacokinetic classes can provide a better understanding of the parameters that assist in the design of orally available, safe and efficacious drugs. A total of thirty ADMET descriptors were computed for each molecule. These include (i) adsorption: water solubility (log mol/l), Caco-2 permeability (log Papp in 10^−6^ cm/s), human intestinal absorption (% absorbed), skin permeability (log Kp), P-gp substrate binding, P-gp I inhibition, and P-gp II inhibition; (ii) distribution: human VDss (log L/kg), human unbound fraction (Fu), blood brain barrier permeability (log BB), and central nervous system permeability (log PS); (iii) metabolism: CYP2D6 substrate binding, CYP3A4 substrate binding, CYP1A2 inhibition, CYP2C19 inhibition, CYP2C9 inhibition, CYP2D6 inhibition, and CYP3A4 inhibition; (iv) excretion: total clearance (log ml/min/kg), and renal OCT2 substrate binding; and (v) toxicity: AMES toxicity, maximum human dose (log mg/kg/day), hERG I inhibition, hERG II inhibition, oral rat acute toxicity (LD50, mol/kg), oral rat chronic toxicity (LOAEL, log mg/kg_bw/day), hepatotoxicity, skin sensitization, *Tetrahymena pyriformis* toxicity (log μg/l), and minnow toxicity (log mM).

### Overview of the StreptomeDB compound card

**Figure 2.**
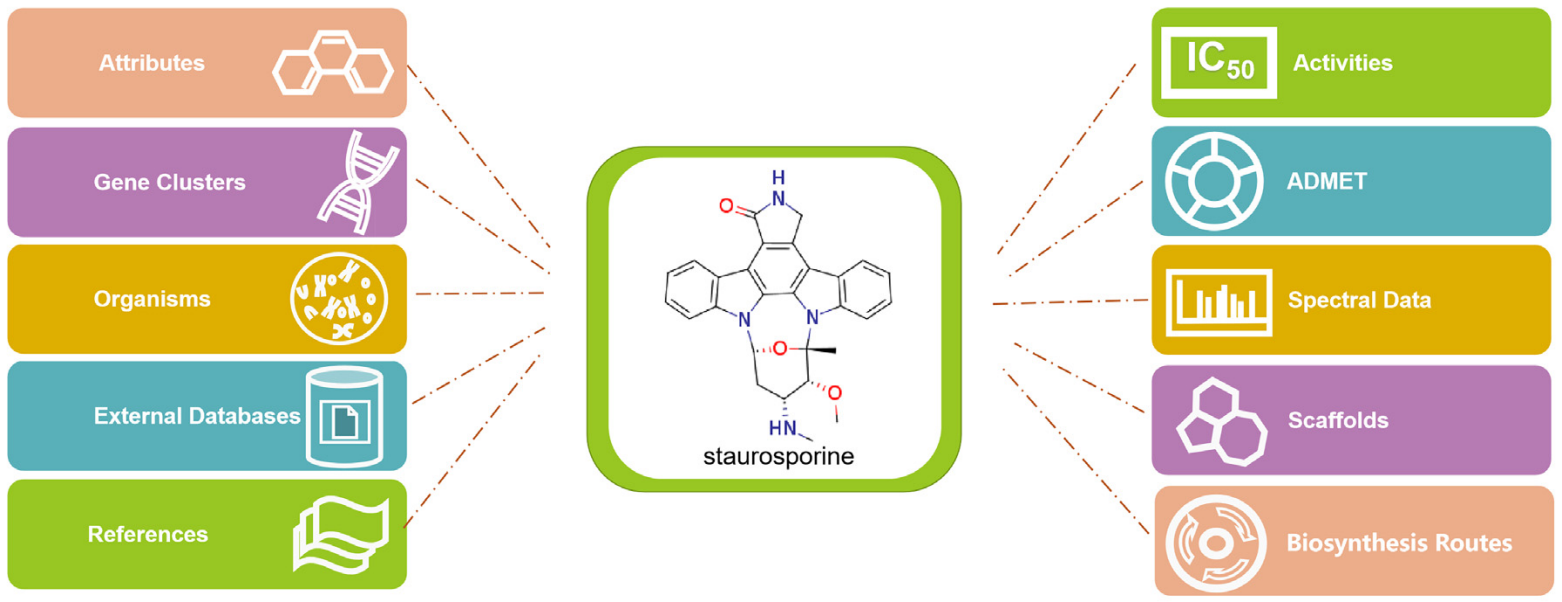
Knowledge graph of the StreptomeDB compound card with staurosporine as example.

The depiction of 2D chemical structures displayed on compound cards (as well as the scaffold browser) of the StreptomeDB web-user interface (WUI) were updated by applying a new version of the command-line tool Molconverter (Marvin 20.18.0, 2020, ChemAxon, https://chemaxon.com/). All new features, namely the hyperlinks and predicted ADMET properties, were added to the compound card. A knowledge graph of the StreptomeDB compound card is shown in Figure 2.

## USE CASES

StreptomeDB WUI and its downloadable data have widely been used in numerous real-world use cases. These can broadly be classified in four categories: (i) data integration in bioinformatics pipelines and other databases, (ii) substructure searching and virtual screening, (iii) structural dereplication, and (iv) comparative data analysis and benchmarking. In the following, we shall highlight some of the most recent real-world use cases, performed since the second release.

### Data integration in bioinformatics pipelines and other databases

The chemical structures of StreptomeDB molecules were used as training data of bioinformatics pipelines such as the NP-likeness scorers, NaPLeS (34) and NP-Scout (35), and the peptidic NP dereplecators, VarQuest (36) and NPS (37). With regards to database integration, the non-ribosomal peptide and macrolactone subsets of StreptomeDB were incorporated into Norine (38) and MacrolactoneDB (39), respectively. Moreover, all StreptomeDB molecules were incorporated in non-redundant NP datasets, such as NPASS (40) and COCONUT (41).

### Substructure searching and virtual screening

In order to identify novel tryptophan 6‐halogenase enzymes, Lee *et al.* used 6‐chloroindole as substructure to query StreptomeDB and retrieved related NPs and their producer organisms (42). From the results, the authors selected and purchased the *Streptomyces albus* N‐16041 strain, from which they later on characterized a novel tryptophan 6‐halogenase enzyme and identified its key catalytic residues for regioselective aromatic halogenation.

StreptomeDB molecules have also been used in both ligand- and structure-based virtual screening. On the one hand, Dias *et al.* developed quantitative structure–activity relationship (QSAR) models for the prediction of antibacterial activity against methicillin-resistant *Staphylococcus aureus* (MRSA) (43). The authors screened StreptomeDB molecules against the best QSAR model and identified 212 hits with predicted minimum inhibitory concentration below the threshold of 5 mM. From these hits, 112 are annotated as antibiotics in StreptomeDB, with four of them as anti-MRSA agents. On the other hand, Kalhor *et al.* docked StreptomeDB molecules to the GyrA subunit of the DNA gyrase enzyme to identify antibiotics that can overcome fluoroquinolone resistance in wild-type and mutated GyrA (44). Three angucycline-possessing hits were identified as potential binders of both wild-type and mutated GyrA.

### Structural dereplication

Mehetre *et al.* matched the experimental MS data of new isolates from *Streptomyces* sp. GH176 to the predicted MS data of StreptomeDB and they dereplicated the chemical structures of abyssomicin I and terpentecin (45). Similarly, Arn *et al.* used the StreptomeDB WUI to dereplicate the structure of dihydropicromycin, isolated from *Streptomyces* sp. W367A (46).

### Comparative data analysis and benchmarking

In a couple of studies (47,48), the druglikeness of StreptomeDB molecules was profiled and compared with that of other NP databases including NANPDB (48) and NuBBE_DB_ (49), and also with that of the DrugBank (21) dataset of experimental/approved drugs. We equally used StreptomeDB molecules as a benchmark dataset for the aforementioned SeMPI web server (32).

## CONCLUSION

Streptomycetes harbor unique and structurally diverse NPs that can serve as leads for the development of various new drugs, notably against antibiotic-resistant bacterial infections. StreptomeDB (http://www.pharmbioinf.uni-freiburg.de/streptomedb) is the first and only public compendium solely dedicated to streptomycetes and their NPs. In this third release, it has grown to contain about 6500 NPs originating from about 3300 producer streptomycetes strains. Moreover, new features were added to StreptomeDB to enhance its usability. These new features include hyperlinks to a number of diverse public online resources. Additionally, we improved the physicochemical descriptions of database molecules by including predicted ADMET properties, and the WUI was updated to enhance user-friendliness. The highlighted real-world use cases clearly demonstrate its wide range of applicability in life sciences. With this increase in the number of entries and in information for each entry, it is hoped that StreptomeDB would become more useful.
